# Mentorship Strategies for New Principal Investigators in Computational Chemistry

**DOI:** 10.1021/acs.jcim.6c01193

**Published:** 2026-06-12

**Authors:** Matheus Ferraz, Tarak Karmakar, Abdurrahman Olğaç

**Affiliations:** † NEC OncoImmunity AS, Oslo Cancer Cluster, Innovation Park, Ullernchausséen 64, 0379, Oslo, Norway; ‡ Department of Chemistry, Yardi School of Artificial Intelligence, 428848 Indian Institute of Technology, Delhi, Hauz Khas 110016, New Delhi, India; § Department of Pharmaceutical Chemistry, Faculty of Pharmacy, 111342 Gazi University, Yenimahalle 06560, Ankara, Türkiye; ∥ Department of Drug Discovery, Evias Pharmaceutical R&D Ltd., Gazi Teknopark, Gölbaşı 06830, Ankara, Türkiye

## Abstract

Computational chemistry has become increasingly central across many sectors, from pharmaceuticals to materials science and beyond. For new principal investigators (PIs), the role extends far beyond technical expertise and includes assembling the right resources and deploying them effectively. Success depends in part on the ability to guide junior researchers with clarity and adaptability, including promoting critical thinking, encouraging collaboration, and cultivating open dialogue grounded in mutual respect. In particular, new PIs must navigate regulations, develop effective research strategies, build collaborations, and recruit emerging talent, while recognizing that each decision shapes outcomes at both the project and career level. In this Viewpoint, we offer insights into mentoring approaches for new PIs, drawing from experiences and observations across academia and industrial settings, with the aim of helping to build the next generation of research leaders.

## Graphical abstract

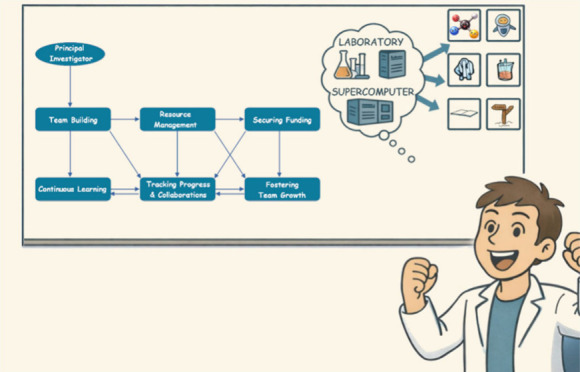

Computational Chemistry (CompChem) has become an integral part of research across diverse sectors, from pharmaceutical discovery and materials design to biomedical research. Recent advances in machine learning potentials, AI-assisted workflows, and cloud-based computing have substantially expanded both the methodological scope and the pace of change within the field. For new principal investigators (PIs), technical expertise must therefore be balanced with responsibilities for team building, resource management, and scientific leadership. Developing hypotheses, systematically testing them, curating and interpreting data, and refining conclusions based on the findings constitute the fundamental workflow of a research group.^1^ Yet, PIs need to decide how to build their research team, access and manage high-performance computing (HPC) resources, secure starting grants, and keep pace with methodological developments in the field. While managing these tasks, the PI should also track ongoing projects and pursue new collaborations across academic and industrial settings. Alongside these activities, effective mentorship has a central role in shaping professional growth and successful careers for all team members, and the team may benefit from working collaboratively on tasks, which can foster critical thinking and collective problem-solving. Such collaborative and mentored environments have been shown to influence intellectual development and increase the likelihood of high-impact scientific achievement.^2^

PIs typically draw on one or more established approaches to develop a mentoring style suited to their group and institutional context. These approaches include traditional senior-junior mentoring; peer-to-peer mentoring, which facilitates expertise exchange among colleagues at similar career stages; and reverse mentoring that helps adapt newly emerging technologies through the contributions of early career researchers or trainees.^3^ Key characteristics of effective mentors identified in the literature include: (i) supporting the mentee’s long-term career development and recognizing that professional relationships often extend past the formal training period and evolve into lifelong connections; (ii) demonstrating enthusiasm and providing intellectual motivation; (iii) showing respect for individual personality traits and remaining sensitive to personal circumstances through attentive listening and empathy; (iv) building confidence and independence in the mentee; (v) supporting a healthy work–life balance; and (vi) offering appreciation and celebrating achievements.^4^

Building connections with peers and broader professional networks is equally important for helping new PIs manage interdisciplinary research environments and sustain long-term scientific growth. Structured team activities can encourage newly established teams’ individual members that have diverse experiences and mentalities to ask questions, develop hypotheses, and learn together.^5^ Such environments also support the development of original research directions rather than strictly adhering to mentors’ opinions and viewpoints. Activities such as journal clubs can further strengthen this process by promoting critical discussion, scientific curiosity, and exposure to emerging ideas across disciplines.

In CompChem, these mentoring principles must be exercised within a technically demanding environment. Effective training, therefore, requires integration across chemistry, biology, physics, mathematics, and computer science, reflecting the increasingly interdisciplinary nature of CompChem applications. Mentorship should also encourage active knowledge sharing through a *balanced learning and teaching model*. Through teaching others, trainees consolidate their understanding and contribute to a stronger team culture. Structured knowledge sharing practices can help team members with diverse backgrounds ask better questions, develop hypotheses, and pursue original research directions independently of the mentor’s own views. As computational chemistry continues to evolve rapidly through advances in AI, AI-driven simulation methods, and large-scale computing infrastructure, it becomes increasingly important for researchers to understand the fundamental chemical and physical principles underlying the tools, methodologies, and algorithms they employ, as well as the computational infrastructure that enables them.

HPC resource management is a practical challenge that new PIs often underestimate. Cloud-based infrastructures improve the accessibility and scalability of various computational workflows, particularly for newly established research groups in CompChem with limited access to dedicated computational resources, enabling large-scale simulations, big data analysis and collaborative projects without requiring major local infrastructure investments.^6^ In addition to securing allocation time, effective resource management requires teaching the team to profile computational costs, optimize job scheduling, and make deliberate trade-offs between accuracy and throughput. Advances in quantum-centric computing paradigms, by integrating quantum processors with current HPC resources, suggest promising future applications in CompChem that will also change the way we store data and simulate, as soon as the tools are easily accessible and mature.^7,8^ The growing accessibility of quantum computing platforms adds an important new dimension to this responsibility. Algorithms such as the Variational Quantum Eigensolver (VQE) and the Quantum Approximate Optimization Algorithm (QAOA) are now accessible through cloud services such as IBM Quantum and Azure Quantum, yet current hardware constraints, particularly qubit count and noise levels, still limit practical application to small molecular systems. New PIs should train their teams to critically evaluate when a quantum approach offers a genuine methodological advantage rather than pursuing it for novelty, and to remain alert to the pace of change in the hardware landscape.

In contrast to the difficulties encountered in assembling the necessary components of research, new technologies can support high-end research when used properly and ethically. Large language models (LLMs), including general-purpose tools such as ChatGPT, Gemini, Claude, and scientific workspaces such as LeapSpace, can generate initial code templates for routine computational tasks, debug scripts, and draft literature summaries for common CompChem tasks. Recent studies have demonstrated that LLMs can assist in literature synthesis and hypothesis generation when used appropriately. Several critical limitations warrant attention: (i) LLMs can generate chemically or physically implausible suggestions with high confidence, requiring expert validation; (ii) they lack a genuine understanding of underlying scientific principles and cannot assess the appropriateness of methods for specific research questions; (iii) training data cutoffs and hallucination tendencies mean they may propagate outdated practices or fabricate references and protocols that appear legitimate. Studies examining LLM performance on chemistry-specific tasks have revealed significant error rates in molecular property prediction and reaction mechanism proposals.^9,10^ A mentor’s role includes guiding appropriate LLM use, demonstrating when these tools add value, when they introduce risk, and how to validate and benchmark their outputs against established chemical knowledge, curated data sets and reference workflows. In addition to standalone LLM usage, emerging AI agent-based systems may further transform CompChem workflows. Such agents can automate literature retrieval, perform molecular design, virtual screening, and downstream data analysis. However, despite their growing capabilities, these systems still require substantial human oversight at every decision point to avoid compounding errors throughout the research pipeline.^11^ Mentors should prepare their teams to understand that automation does not transfer scientific responsibility away from the researcher. Data privacy constraints must be explicitly addressed when integrating cloud-based or public AI tools, particularly when proprietary or clinical data are involved. These considerations become even more pressing as AI components are embedded within a broader automated research platform.

Interdisciplinary and collaborative environments may increasingly involve integrated translational design and discovery frameworks that combine CompChem, AI, laboratory automation, robotics, and experimental sciences to accelerate the translation of fundamental scientific discoveries into practical applications. Integrated platforms combining robotics, automated synthesis, high-throughput experimentation, AI-guided design, and real-time analytical feedback are enabling partially autonomous design–make–test–analyze (DMTA) cycles. Such systems may substantially accelerate chemical optimization, reduce experimental bottlenecks, and improve reproducibility by integrating computational and experimental workflows into unified digital infrastructures. Recent industrial initiatives, including highly automated laboratory platforms developed in chemical R&D environments, illustrate how automation, AI, and robotics may increasingly operate together within future chemical discovery pipelines,^12^ and make reproducibility an operational necessity.^13^

Reproducibility is a structural habit that must be built into a research group from its earliest days. The JCIM editorial by Merz and co-workers established a community-wide expectation that authors document how their data and software can be accessed, recognizing that conclusions dependent on undisclosed code or proprietary data sets cannot be independently verified or extended.^14^ Soares and co-workers subsequently extended these expectations to molecular dynamics specifically, highlighting that reporting replica simulations and convergence criteria is essential for assessing the reliability of results.^15^ New PIs should institutionalize these standards within their groups by requiring version-controlled repositories using Git on platforms such as GitHub or GitLab, reproducible computational environments specified through conda or container tools such as Docker, and data sets archived with persistent identifiers on repositories such as Zenodo or Figshare. Structured project directories that allow a new team member or external collaborator to reproduce a result without consulting the original author are not an overhead but a form of scientific accountability. These practices also offer practical benefits apart from compliance. They reduce knowledge loss during team turnover, simplify onboarding, and make collaborative projects substantially more tractable. In industrial settings, reproducibility takes a different form, as workflows must be fully documented and traceable for internal audit and regulatory purposes. However, the underlying data and proprietary models are often not publicly disclosed, creating a tension between scientific transparency and intellectual property protection. This tension is becoming increasingly important as modern AI-driven drug discovery workflows rely heavily on large-scale, well-curated, and reproducibly accessible data sets and algorithms, prompting growing calls within the cheminformatics community for FAIR and openly shareable research infrastructures.^16^

Although publication remains a major milestone in academia, effective mentorship extends far beyond guiding a team toward scientific papers. Instead, PIs should aim to equip their group members with long-term skills, including critical thinking, a deep and adaptable understanding of scientific methods and research areas, and the ability to communicate and collaborate across disciplines. Furthermore, PIs should actively encourage mentees to participate in conferences and workshops, and professional training programs, where they can present their work and engage with the broader scientific community. Effective mentorship in this context includes helping trainees craft compelling abstracts, rehearsing presentations with constructive feedback, and debriefing after conferences to consolidate networking connections and identify collaborative opportunities. Over time, these interactions may develop into broader interdisciplinary collaborations and consortium-based projects addressing major societal challenges in areas such as energy, environmental sustainability, and human health. Importantly, the progression toward such opportunities is rarely linear, and mentorship should therefore prepare trainees to pursue various paths and make informed decisions in planning and executing their research careers.

Failure and rejection, including manuscript desk rejections, unsuccessful grant applications, or failed investment efforts, irreproducible simulation results, failed benchmarking studies, or unsuccessful virtual screening campaigns, are inevitable aspects of scientific research and can be particularly discouraging to early career researchers. PIs therefore need to normalize these experiences, guide mentees constructively, and encourage them to learn from such setbacks. Sharing personal experiences of failures fosters authenticity and breaks down the stigma around setbacks. Preparing trainees to handle rejection is itself a form of mentorship with lasting career impact. Empathy is central to this process, building trust and psychological safety that enables mentees to take intellectual risks and develop with confidence. These interpersonal dimensions of mentorship become particularly acute in industrial settings, where external pressures add layers of complexity.

Particularly for those working in or alongside industry, while foundational scientific innovation remains important, PIs in these settings must also operate within additional constraints and expectations. These include aligning research priorities with business strategies, adhering to regulatory standards, and responding to compressed project timelines. Research and development in industry frequently involve complex organizational structures, contractual obligations, and interdisciplinary collaboration across departments with competing priorities. New PIs should mentor their teams to balance computational innovation with practical deliverables by establishing clear project milestones, holding regular progress discussions, and helping junior researchers recognize when perfectionism may hinder progress. Early career PIs should develop competence in managing nondisclosure agreements (NDAs), crafting publication strategies that respect proprietary boundaries, and working with external partners while protecting sensitive data. For instance, a PI developing machine learning models for lead optimization may need to publish methodological innovations while protecting the specific molecular scaffolds and structure–activity relationships that constitute trade secrets. Rather than simply enforcing rules, effective mentors help trainees develop judgment about disclosure timing and collaboration agreements. Understanding how to deal with internal disclosure and patent filing processes enables researchers to protect innovation without unnecessarily delaying scientific dissemination. Regulatory submissions increasingly rely on computational outputs, including toxicology risk assessments and pharmacokinetic models, requiring teams to follow data handling practices in line with FAIR principles and agency guidance. Ethical awareness, especially in areas involving clinical or genomic data, should also be part of early mentorship, ensuring that computational researchers understand data privacy regulations, informed consent implications, and responsible use of sensitive data sets.

Fulfilling these responsibilities across diverse teams and shifting priorities demands deliberate approaches to project management. Various approaches can support these challenges, including agile project management, which provides practical frameworks and tools. New PIs can use project management frameworks not just for organization, but as mentorship tools. Regular check-ins become opportunities to identify when trainees are stuck, retrospective meetings normalize learning from failed calculations, and breaking goals into tasks teaches project planning skills trainees will use throughout their careers. Agile project management provides flexible frameworks, short work cycles, clear deliverables, and frequent progress reviews. In practice, this involves breaking research goals into manageable tasks, tracking them with tools like Kanban boards, and conducting regular check-ins to address obstacles early. Such habits encourage transparency, improve coordination, and allow teams to adapt as priorities change. For new PIs, leading with agility means aligning research milestones with evolving experimental or business needs while maintaining scientific standards. Developing these habits early can greatly enhance a PI’s ability to integrate computation into fast-paced research environments. Though agile has gained popularity in industry-facing computational chemistry for its focus on responsiveness, it is important to understand that no single management style fits all projects. Effective leadership relies on choosing and adapting methods that suit the specific scientific and organizational context.

It is worth noting that early career mentors also continue to grow alongside their mentees. This reciprocal process of growth increases the likelihood that successful mentors will cultivate successful mentees, a relationship supported by empirical studies. At the same time, it must not be overlooked that true success becomes evident when the mentee begins to produce independent work. Ultimately, the journey of new PIs in CompChem is defined by continuous learning, adaptation, and the careful balancing of scientific, technical, and interpersonal responsibilities. Building a research environment that values curiosity, resilience, inclusivity, and collaboration is essential for sustaining long-term scientific growth. New PIs can thrive in a rapidly evolving landscape, a scenario where CompChem continues to shape solutions for some of society’s most urgent challenges by merging deep technical knowledge, careful team development, strategic resource management and ethical responsibility.
